# Disrespect and abuse during childbirth in Western Ethiopia: Should women continue to tolerate?

**DOI:** 10.1371/journal.pone.0217126

**Published:** 2019-06-07

**Authors:** Firew Tekle Bobo, Habtamu Kebebe Kasaye, Belachew Etana, Mirkuzie Woldie, Tesfaye Regassa Feyissa

**Affiliations:** 1 Department of Public Health, Wollega University; Nekemte, Oromia, Ethiopia; 2 Department of Midwifery, Wollega University; Nekemte, Oromia, Ethiopia; 3 Department of Health Policy and Management, Jimma University; Jimma, Oromia, Ethiopia; 4 Fenot Project, Harvard T.H. Chan School of Public Health, Department of Global Health and Population, Addis Ababa, Ethiopia; 5 Research Centre for Generational Health and Ageing, Faculty of Health and Medicine, The University of Newcastle, Newcastle, Australia; Public Health Foundation of India, INDIA

## Abstract

**Background:**

Healthcare coverage in Ethiopia has improved dramatically in recent decades. However, facility-based delivery remains persistently low, while maternal mortality remains high. This paper presents the prevalence and associated factors of disrespect and abuse (D&A) during childbirth in public health facilities of western Oromia, Ethiopia.

**Method:**

A facility-based cross-sectional study was conducted among 612 women from February 2017 to May 2017. Exit interview with the mothers were conducted upon discharge from the maternity ward. We measured D&A during childbirth using seven dimensions. Multivariable logistic regression model was used to assess the association between experience of D&A and client characteristics and institutional factors.

**Result:**

Three quarters (74.8%) of women reported experiencing at least one form of D&A during their facility childbirth. The types of D&A experienced by the women were; physical abuse (37.1%), non-dignified care (34.6%), non-consented care (54.1%), non-confidential care (40.4%), neglect (25.2%), detention (2.9%), and discrimination (13.2%). Experiences of D&A were 1.6 times more likely to be reported by women delivering at hospitals than health centers (OR: 1.64, 95% CI: 1.01, 2.66). Women without a companion throughout their delivery were almost 10 times more likely than women who had a companion to encounter D&A (OR: 9.94, 95% CI: 5.72, 17.28). On the other hand, women with more than 1,368-birr (USD 57) monthly income were less likely to experience any type of D&A (OR: 0.36, 95% CI: .21, .65).

**Conclusion:**

Three in four women reported experiencing at least one form of D&A during labor and delivery. This demonstrates a real disconnect between what the health system intends to achieve and what is practiced and calls for fundamental solutions in terms of both improving quality of facility-based delivery and ensuring women’s right to receive health care with dignity.

## Background

Access to health care facilities has dramatically improved over the past two decades in Ethiopia. However, utilization of maternal health care services has remained considerably low [1–4]. The women who do attend health facilities sometimes lack access to respectful and caring maternal health services [5–7]. Experiencing or fearing encountering abuse and humiliation by healthcare professionals during labor and delivery can deter women from using health care facilities during subsequent deliveries [8, 9]. Other studies from sub-Saharan African countries have reported high levels of disrespectful and abusive maternity care during childbirth. Prior studies documented up to 98% of women in Enugu, southeastern Nigeria [10], and 78% of women in Addis Ababa, Ethiopia [11], faced at least one form of D&A during childbirth, and 58.2% of women lacked privacy during labor and delivery in Malawi (direct observation) [12].

Women in low-income countries may face problems when trying to access safe and respectful childbirth care [13, 14]. Before reaching the health facility, they may encounter cultural barriers to seeking care, long distance and lack of transport to go to the health facilities, and indirect costs of service even when direct care is free [13, 15, 16]. Upon entering the health facility, they can be faced with overloaded health facilities which are understaffed and have insufficient drugs and supplies [14, 17]. This is worsened when there is dehumanization and abuse during childbirth [18]. As a result, women become discouraged to seek care at the health facilities and hence, might not come back if pregnant again, potentially decreasing women’s attendance at health facilities for childbirth with increasing birth order [7, 19, 20].

The 2016 Ethiopian Demographic and Health Survey demonstrates the continued low utilization of maternal health services; 32% of women receive four antenatal care (ANC) visits and 26% of women deliver at a health facility, with only 17% of mothers receiving postnatal care within two days of birth [3]. Such underutilization of care from a skilled provider along the maternity continuum may increase the risk of maternal and child mortality from preventable causes.

Disrespectful and abusive maternity care is one of the main reasons for underutilization of maternal health services [20–22]. Several factors can contribute to disrespectful and abusive maternity care. In resource-limited settings, established patterns of low quality and undignified care has been accepted as normal in local cultures, making disrespectful care less recognized as being a problem [5, 23–25]. There is also a lack of community engagement in health governance in these health care systems, thus limiting accountability of providers to their clients [5]. Moreover, health care professionals in low-income settings usually face poor working conditions that may cause poor motivation, burnout, frustration, and demoralization [26]. All of these are commonly experienced in overloaded health care systems such as Ethiopia and contribute to the mistreatment and abuse of patients by providers [7, 8, 22].

The Ethiopian Health Sector Transformation Plan (HSTP) (2015/16–2019/20) intends to reduce the current maternal mortality ratio (MMR) of 420 to 199/100,000 and neonatal mortality rate of 29 to 10/1000 [27]. As a means to reach this target, the plan specifies improving quality of health services by transforming how healthcare providers treat patients and clients. The caring, respectful and compassionate (CRC) health workforce initiative in this plan intends to address the concern of D&A for clients, including laboring mothers [27]. However, there is a lack of empirical evidence and key indicators on the actual burden of the problem in the country, except for a few reports published years ago [7, 11, 24]. Hence, the results from this study are timely to influence the policy implementation process by providing evidence of how the health workforce treats women coming for delivery services in public health facilities. This study aims to examine the prevalence and associated predictors of D&A as reported by women during labor and delivery in public health facilities of western Oromia region in Southwestern Ethiopia.

## Methods

### Study design and setting

A facility-based cross-sectional study was conducted from February, 2017 to May, 2017. The health service delivery system in Ethiopia is structured in a three-tier system, which includes primary, secondary and tertiary levels of health care, linked together for referral. The primary level serves up to 100,000 population. The secondary level handles up to 1.5 million people, while the tertiary level provides service for 3.5–5 million population. The primary level of the health care system–the primary health care unit (PHCUs) -is composed of a primary hospital, capable of providing both basic emergency obstetric care (BEmOC) and comprehensive emergency obstetric and neonatal care (CEmONC), health centers that can provide basic emergency obstetric care, and, their satellite health posts that work on providing health information, identifying pregnant women and referring them to health centers for further follow-up. The secondary level of the health system includes general hospitals, while the tertiary level is composed of specialized hospitals—both capable of providing comprehensive emergency obstetric and neonatal care [27, 28].

This study was conducted in three zones of western Oromia that includes East Wollega, West Wollega and Kellem Wollega. In the study area, there were 9 public primary hospitals and 2 missionary hospitals, 182 health centers, 1041 health posts and 4 general hospitals. During the data collection period, each of the health centers included in the study had one delivery bed and one delivery care provider (midwife or nurse), while the hospitals had 2 to 3 delivery beds with a maximum of three delivery care providers.

### Study participants

Data was collected from three public hospitals and three public health centers—one hospital and one health center from each zone. Women who delivered at the selected health facilities during the study period (February/2017 to May/2017) were interviewed upon discharge (exit interview). Single population proportion formula was used to estimate the sample size. With the assumptions of 95% confidence interval, 0.03 precision, and 10% non-response rate, and using the proportion of women who faced at least one form of D&A from the previous study in Addis Ababa (78%) [11]. The final calculated sample size was 807. The sample was allocated proportionally to the selected health facilities based on the facility delivery reports from the previous one-year.

### Data collection procedures

Data collectors approached women upon discharge from the maternity ward after giving birth to their child. Ten data collectors, who were female nurses holding a BSc. degree and not affiliated with the study facilities, conducted the interviews using a semi-structured questionnaire. A three-day training session was provided for data collectors before commencing data collection to ensure full understanding of the study objectives, data collection instruments, and informed consent procedures. The data collectors described the nature and objectives of the study, and obtained the consent of the women for participation in the study. All interviews were conducted in a private room at the health facilities to ensure confidentiality and privacy.

### Measurement

The items in the questionnaire were adapted and developed after a thorough review of the literature [5, 6, 10, 11, 29–31]. The questionnaire had three components that included socio-demographic characteristics, the most recent history of healthcare utilization, perceived quality of health care services and experiences of disrespectful and abusive maternity care during childbirth. The survey questionnaire and the consent form were first developed in English, translated into Afaan Oromo (the dominant language spoken in Oromia region), and then back-translated to check for semantic equivalence.

The outcome variable of the study was the subjective experience of D&A among women who had given birth at the selected health facilities. We measured D&A using a framework developed by Bowser and Hill [5]. This framework was further adapted to the local context and types of D&A were then categorized as physical abuse, non-dignified care, abandonment, non-consented care, non-confidential care, detention and discrimination. These categories were then further extended to capture the particular events and specific forms of D&A as shown in Table 1. Questions addressing each of the different forms of D&A were posed to the respondents during the exit interview. If the women have experienced any type of D&A, then they were asked to describe the nature and extent of D&A. In each category of D&A, after responding to the close-ended questions, women were asked to describe if there was any other event, they considered disrespectful and abusive. The close ended questions were identified and derived from extensive review of literature [5, 6, 10, 24, 30, 31]. For example, the close ended questions under physical abuse were posed as follows; “At any point during your stay for this delivery, were you slapped, beaten or pinched?” This type of specific questioning was used for all of the categories of D&A, in order to be clear about what constituted each type of D&A. However, under the discrimination category, open ended questions were posed to the women who answered affirmatively. For the discrimination category, women were asked the following question; “At any point during your stay for this delivery, were you discriminated/felt discriminated against by any of the healthcare workers?” If a woman responded “yes” to this question, then she was asked to describe the nature of the discrimination. The data acquired through descriptive documentation were then categorized and integrated under each kind of D&A during data cleaning.

**Table 1:** Categories and sub-components of disrespect and abuse.

Categories of D&A	Forms of D&A
Physical abuse	Hitting (slapped, beaten or pinched), harshly forcing legs apart, tied down during labor
Non-dignified care	Shouted at, threat of withholding treatment, blamed or intimidated
Non-consented care	Non-consented episiotomy, C-section, tubal ligation
Non-confidential care	Provision of care without privacy, medical history disclosed without consent
Neglect/ abandonment	Gave birth outside delivery room (corridor, waiting room or floor), ignored when needed help, delivered without skilled attendant
Detention	Detention in health facility for failure to pay, request for bribe
Discrimination	Denial of needed attention on the basis on areas of residence (urban/rural), denial of needed attention on the basis of age (old/young), denial of needed attention on the basis of occupation/education

### Data processing and analysis

Data was first cleaned and entered into Epi-info (V-3.5.2) and then exported to STATA 13 (College Station, TX, USA) for further analysis. Descriptive statistics were computed and presented, using frequencies and proportions for categorical variables, and summarized by mean and standard deviation for continuous variables. Frequencies of D&A components and subcomponents were also computed and reported in a table. The key outcome of the study was any form of D&A experienced by delivering women. The specific categories of D&A (physical abuse, non-dignified care, non-consented care, non-confidential care, abandonment, and discrimination) were also considered as outcomes of this study.

The independent variables of interest for reported D&A included socio-demographic characteristics (age, marital, education, occupation, area of residence, family annual income), type of health facility (health center and hospital), and delivery characteristics (number of children alive, use of ANC, time of delivery, number of providers, sex of providers and presence of a labor companion).

To identify predictors of the seven D&A categories we first conducted bivariate regressions with each potential covariate, and the decision of which covariates would be included in the final multivariable model was made based on P < 0.05. Then we fitted seven multivariable logistic models (one model for each category of D&A, except detention), with a 95% confidence interval. Detention was excluded as we could not perform further statistical analyses because the number of women who reported detention was too small. Results were then presented as adjusted odds ratios (OR). All statistical analysis was performed using STATA 13.

### Ethical considerations

Ethical clearance was obtained from Wollega University ethical review committee and written permission letters from the three zonal health bureaus were granted. Administrations of the selected health centers and hospitals were informed of the study objectives and protocols.

The ethical review committee of Wollega University approved verbal consent because nearly half of the women in the country never attended school [3]. The purpose and process of the study were explained to all participants. They were informed that their participation was voluntary and that they could withdraw at any time for any reason without any penalty either personnel or affecting their future medical care. The verbal consent was obtained by asking the women if she would like to participate in the study after explaining the purpose and reassuring her of the confidentiality of the survey. If the women responded “yes” then the data collectors ticked on the "yes" box of the consent form on the questionnaire, and then the women were interviewed. If the women responded “No” then the data collectors ticked the “no” box of the consent form and women were excluded from the study. For study participants aged less than 18, we received verbal informed consent from the respondents themselves, as woman under 18 who are married are considered to be “mature minors” and are able to provide consent. The ethics committee approved the consent procedure for all including aged less than 18 years.

Contact details of the study coordinator was provided to the participants for any questions or concerns. No identifiers were used in the analysis to ensure confidentiality.

## Results

### Socio-demographic characteristics of the respondents

Among 807 invited women, 612 women who gave birth to their child at the selected health facilities participated in the study (76% response rate). The majority of participants were married (94%(N = 575)), were ethnically Oromo (86.1% (N = 527)), followed the protestant religion (53.8% (N = 329)), were college or University level educated (29.7% (N = 182)), were between the age group of 25–29 years (42.2% (N = 256)), were from urban areas (69.9% (N = 428)), and were housewives (61.0% (N = 373)). Regarding maternal health service utilization, among the 612 interviewed women, the majority (39.7% (N = 243)) had just given birth to their first child, 97.5% (N = 597) had received antenatal care (ANC), 74.2% (N = 454) of the respondents gave birth at hospital, and 55.6% (N = 340) had a companion with them during labor and delivery (Table 2).

**Table 2:** Socio-demographic and service utilization characteristics of the respondents (n = 612).

Socio-demographic characteristics	Frequency	Percent
**Marital Status**		
Single	13	2.1
Married	575	94.0
Divorced	14	2.3
Widowed	10	1.6
**Educational Status**		
No Formal Education	91	14.9
Primary school	161	26.3
Secondary School	178	29.1
College/University	182	29.7
**Occupational Status**		
Gov′t employee	147	24.0
Merchant	92	15.0
Housewife	373	61.0
**Age (25.57±4.55)**		
15–19	43	7.0
20–24	196	32.0
25–29	258	42.2
30–34	86	14.1
35+	29	4.7
**Residence**		
Rural	184	30.1
Urban	428	69.9
Monthly household income*		
Less than 1,368-birr	540	88.2
≥1,368-birr	72	11.8
**Number of living births**		
First birth	243	39.7
Second birth	192	31.4
Three and more birth	177	28.9
**History of ANC visit for the last pregnancy**		
No	15	2.5
Yes	597	97.5
**Number of ANC visit**		
1 visit	20	3.3
2 visits	83	13.6
3 visits	185	30.2
4 visits	309	50.5
**Place of delivery**		
Hospital	454	74.2
Health Center	158	25.9
**Number of delivery attendants**		
One	87	14.2
Two	251	41.0
Three to four	208	34.0
Five and above	66	10.8
**Sex of delivery attendants**		
Male	165	27.0
Female	117	19.1
Both male and female attendants	330	53.9
**Presence of labor companion**		
No	272	44.4
Yes	340	55.6

*1 USD was equivalent to 24 birrs during the study period and then multiplied by poverty line income (1.9/day).

### Prevalence of disrespect and abuse

The majority of respondents (74.8%, 95% CI: 71.3, 78.2) reported facing at least one form of D&A during their delivery care (Table 3). Non-consented care was found to be the most prevalent category of D&A (54.1%, 95% CI: 50.1, 58.0). Among subcomponents of non-consented care, unconsented episiotomy was found to be the most common form of non-consented care (48.9%, 95% CI: 44.9, 52.8), defined as the healthcare provider doing the procedure without obtaining informed consent from the client at any time. Non-confidential care was the other most common type of D&A. Two hundred and forty-seven (40.4%, 95% CI: 36.5, 44.3) women reported confidentiality of care was breached, and of these 39.9% (95% CI: 36.0, 43.8) experienced lack of privacy during labor and delivery, while (7.2%, 95% CI: 5.3, 9.4) women reported that their medical history was disclosed by the healthcare professionals without their consent. Physical abuse was the third most common form of D&A, with 37.1% (95% CI: 33.3, 41.0) of women reporting physical abuse. Harshly forcing the legs apart was the most common form of physical abuse (22.2%, 95% CI: 30.2, 37.6). Exposure to non-dignified care was reported by 34.6% (95% CI: 30.9, 38.5) of women. Nearly one-third of all women (31.4%, 95% CI: 27.8, 35.1) said that health care professionals shouted at them during labor and delivery.

**Table 3:** Experiences of disrespect and abuse during childbirth among women from six health facilities in the Western Oromia, Ethiopia, 2017 (N = 612).

Types of D&A	Frequency	Percent	Confidence interval (95%)	
			Lower	Upper
**Any form of D&A**	**458**	**74.8**	**71.3**	**78.2**
**Physical abuse**	**227**	**37.1**	**33.3**	**41.0**
Hitting (slapped, beaten or pinched	136	22.2	19.1	25.7
Harshly forcing leg apart	207	33.8	30.2	37.6
Tied down during labor	10	1.6	0.8	2.9
**Non-dignified care**	**212**	**34.6**	**30.9**	**38.5**
Shouted at	192	31.4	27.8	35.1
Threat of withholding treatment	84	13.7	11.2	16.6
Blamed or intimidated	105	17.2	14.3	20.3
**Non-consented care**	**331**	**54.1**	**50.1**	**58.0**
Unconsented episiotomy	299	48.9	44.9	52.8
Unconsented C-section	32	5.2	3.7	7.2
**Non-confidential care**	**247**	**40.4**	**36.5**	**44.3**
Provision of care without privacy	244	39.9	36.0	43.8
Medical history disclosed without consent	44	7.2	5.3	9.4
**Neglect/ abandonment**	**154**	**25.2**	**21.8**	**28.7**
Gave birth outside delivery room (corridor, waiting room or floor)	107	17.5	14.6	20.7
Ignored when needed help	140	22.9	19.7	26.3
Delivered without skilled attendant	7			
**Detention**	**18**	**2.9**	**1.8**	**4.5**
Detention in health facility for failure to pay	18	2.9	1.8	4.5
Inappropriate demands for payment	0	0		
**Discrimination**	**81**	**13.2**	**10.7**	**16.1**
Denial of needed attention on the basis of low social class	49	8.0	6.0	10.4
Denial of needed attention on the basis of age young (less than 19 years old)	29	4.7	3.3	6.7
Denial of needed attention on the basis of residence area (rural)	40	6.5	4.8	8.7

Abandonment during labor and delivery was also reported frequently. One hundred and fifty-four women (25.2%, 95% CI: 21.8, 28.7) reported that they felt neglected and abandoned while admitted for delivery care. The most commonly encountered subcomponent of neglect/abandonment reported was being ignored when needing help, (22.9%, 95% CI: 19.7, 26.3) followed by giving birth outside of the delivery room (corridor, waiting room or floor), (17.5%, 95% CI: 14.6, 20.7). Table 3 shows the prevalence of D&A by category and subcomponent, and Table 4 shows D&A by socio-demographic characteristics.

**Table 4:** Disrespect and abuse by socio-demographic characteristics, Western Ethiopia, 2017.

Variables	Total (N)	Any type of D&A N (%)	Physical abuse N(%)	Non-dignified N(%)	Non-consented N(%)	Non-confidential N(%)	Neglect/ Abandonment N(%)	Discrimination N(%)
**Birth Order**		**p = 0.171**	**p = 0.003**	**p = 0.330**	**p = 0.207**	**p = 0.000**	**p = 0.473**	**p = 0.608**
First birth	243	191(78.6)	70(28.80)	78(32.1)	142(58.4)	71(29.2)	55(22.6)	29(11.9)
Second birth	192	136(70.8)	81(42.18)	65(33.85)	97(50.5)	92(47.9)	53(27.6)	25(13.0)
Third birth & above	177	131(74.0)	76(42.93)	69(38.98)	92(52.0)	84(47.5)	46(26.0)	27(15.3)
**Educational status**		**p = 0.003**	**p = 0.001**	**p = 0.254**	**p = 0.003**	**p = 0.000**	**p = 0.000**	**p = 0.089**
No Formal Education	91	75(82.4)	46(50.5)	35(38.5)	63(69.2)	52(57.1)	32(35.2)	15(16.5)
Primary	161	105(65.2)	44(27.3)	46(28.6)	74(46.0)	37(23.0)	20(12.4)	12(7.5)
Secondary School	178	131(73.6)	61(34.3)	62(34.8)	90(50.6)	69(38.8)	49(27.5)	27(15.2)
College/University	182	147(80.8)	76(41.8)	69(37.9)	104(57.1)	89(48.9)	53(29.1)	27(14.8)
**Occupation**		**p = 0.299**	**p = 0.001**	**p = 0.206**	**p = 0.018**	**p = 0.001**	**p = 0.114**	**p = 0.436**
Government employee	147	115(78.2)	61(41.5)	57(38.8)	92(62.6)	75(51.0)	43(29.3)	19(12.9)
Merchant	92	72(78.3)	48(52.2)	36(39.1)	54(58.7)	43(46.7)	28(30.4)	16(17.4)
Housewife	373	271(72.7)	118(31.6)	119(31.9)	185(49.6)	129(34.6)	83(22.3)	46(12.3)
**Monthly income**		**p = 0.000**	**p = 0.000**	**p = 0.018**	**p = 0.000**	**p = 0.000**	**p = 0.000**	**p = 0.005**
Less than 1,368-birr	540	422(78.1)	216(40.0)	196(36.3)	306(56.7)	245(45.4)	152(28.1)	79(14.6)
≥1,368 birr	72	36(50.0)	11(15.3)	16(22.2)	25(34.7)	2(2.8)	2(2.8)	2(2.8)
**Presence of companion during delivery**		**p = 0.000**	**p = 0.000**	**p = 0.000**	**p = 0.000**	**p = 0.000**	**p = 0.000**	**p = 0.000**
No	272	254(93.4)	160(58.8)	126(46.3)	183(67.3)	191(70.2)	102(37.5)	62(22.8)
Yes	340	204(60.0)	67(19.7)	86(25.3)	148(43.5)	56(16.5)	52(15.3)	19(5.6)
**Health facility at delivery**		**p = 0.002**	**p = 0.001**	**p = 0.000**	**p = 0.167**	**p = 0.027**	**p = 0.000**	**p = 0.031**
Hospital	454	354(78.0)	186(41.0)	185(40.7)	253(55.7)	195(43.0)	131(28.9)	68(15.0)
Health Center	158	104(65.8)	41(25.9)	27(17.1)	78(49.4)	52(32.9)	23(14.6)	13(8.2)
**Number of delivery attendants**		**p = 0.025**	**p = 0.462**	**p = 0.002**	**p = 0.535**	**p = 0.119**	**p = 0.024**	**p = 0.106**
Two and less	338	241(71.3)	121(35.8)	99(29.3)	179(53.0)	127(37.6)	73(21.6)	38(11.2)
More than two	274	217(79.2)	106(38.7)	113(41.2)	152(55.5)	120(43.8)	81(29.6)	43(15.7)

**Fig 1:**
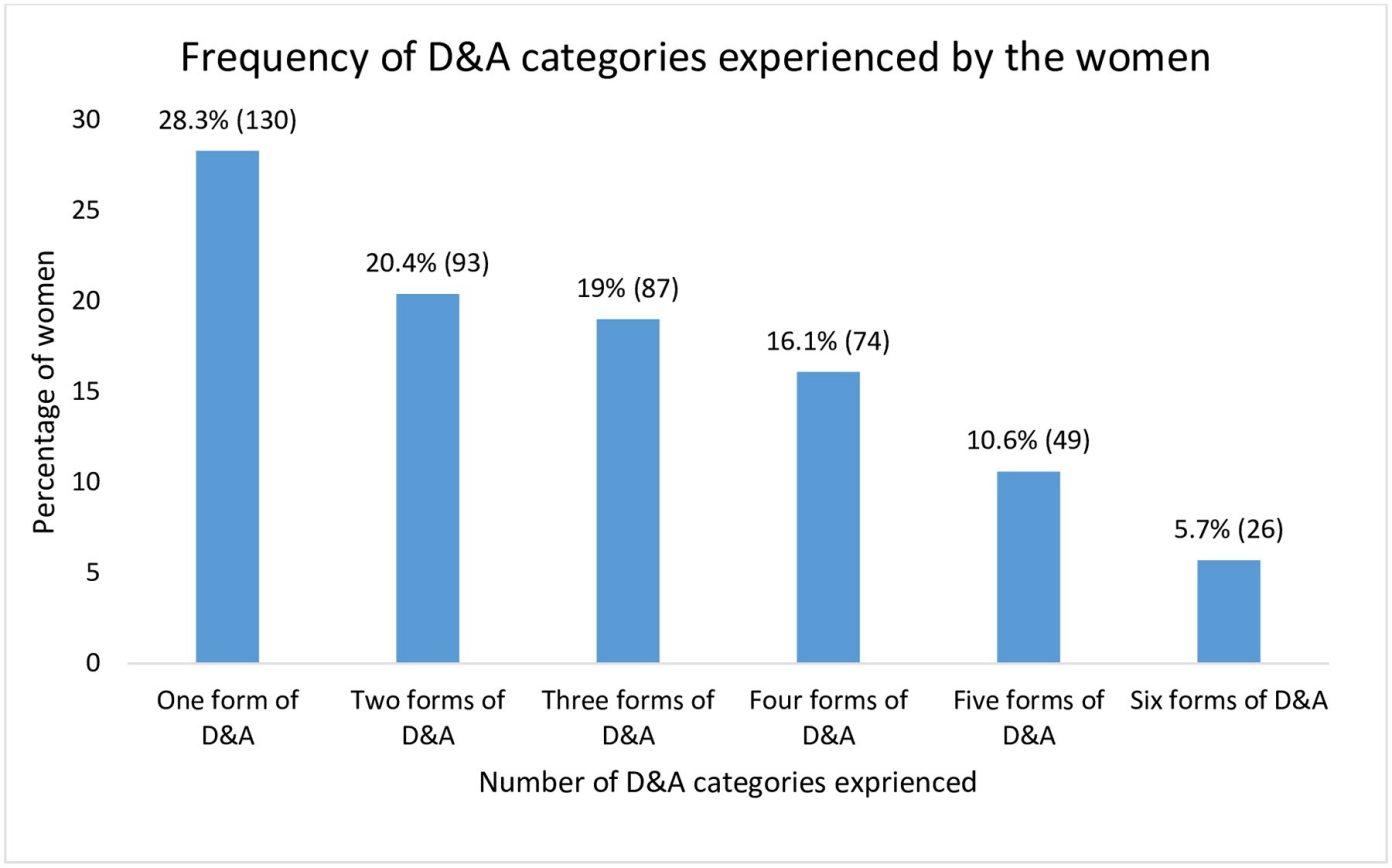
Frequency of D&A categories experienced by the women during child birth (N = 458).

Among mothers who reported experiences of D&A, more than a quarter (28.3%, N = 130) reported one form of D&A, 20.4% (N = 94) reported experiencing two of the categories, and 19.0% (N = 87) reported experiencing three forms of D&A during their delivery care (Fig 1).

### Predictors of D&A

Seven logistic regression models were constructed to examine the relationship of client and institutional characteristics against each of the 7 different categories of D&A. One form (detention) was excluded since bivariate analysis revealed that further statistical analyses could not be performed due to the number of women exposed to this category being too small (Table 5). The presence of a companion during labor and delivery, monthly household income and the type of health facility at which childbirth takes place were found to be significantly associated with most of D&A categories. Lack of a companion during labor and delivery was found to be the most important statistically significant predictor of all forms of D&A, with those without a companion almost 10 times more likely to experience disrespect or abuse (OR = 9.94, 95% CI: 5.72, 17.28). Women who gave birth at a hospital were also significantly more likely to experience D&A than women who gave birth at a health center, with women delivering in a hospital 1.6 times more likely to experience any type of D&A than women delivering in health centers (OR = 1.64, 95% CI: 1.01, 2.66). Women who had given birth to their first child were about two times more likely to report any type of D&A compared to those who had three or more delivery experiences (OR = 1.95, 1.14, 3.34). On the other hand, higher income (more than 1,368-birr monthly) was protective of D&A (OR = 0.36, 95% CI: 0.21, 0.65).

**Table 5:** Predictors of disrespect and abuse during childbirth among women served at health facilities in Western Ethiopia, 2017.

Variables with categories	Any type of DA OR(CI)	Physical abuse OR(CI)	Non-dignified OR(CI)	Non-consented OR(CI)	Non-confidential OR(CI)	Neglect/ Abandonment OR(CI)	Discrimination OR(CI)
**Birth Order**							
3 & above birth	**1**	**1**	**1**	**1**	**1**	**1**	**1**
First birth	1.95(1.14, 3.34) *	0.72(0.45, 1.16)	0.81(.52, 1.27)	1.90(1.23, 2.95) **	0.54(0.32, 0.91) *	1.07(0.64, 1.79)	0.93(0.49, 1.75)
Second birth	0.76(0.44, 1.32)	1.01(0.63, 1.62)	0.72(0.46, 1.17)	1.03(0.66, 1.61)	1.03(0.60, 1.74)	1.13(0.68, 1.89)	0.79(0.42, 1.49)
**Educational status**							
**College/University**	**1**	**1**	**1**	**1**	**1**	**1**	**1**
No Formal Education	0.99(0.37, 2.62)	1.46(0.68, 3.15)	0.99(0.48, 2.08)	3.83(1.83, 8.01) ***	1.95(0.83, 4.58)	1.41(0.65, 3.08)	0.69(0.267, 1.811)
Primary	0.54(0.24, 1.24)	0.90(0.45, 1.81)	0.97(0.50, 1.88)	1.62(0.85, 3.08)	0.77(0.35, 1.66)	0.53(0.24, 1.13)	0.54(0.21, 1.38)
Secondary School	0.53(0.24, 1.17)	0.90(0.47, 1.73)	1.11(0.60, 2.06)	1.44(0.78, 2.62)	1.07(0.53, 2.18)	1.14(0.59, 2.22)	0.88(0.39, 1.97)
**Occupation**							
**Housewife**	1	**1**	**1**	**1**	**1**	**1**	**1**
Government employee	0.61(0.27, 1.37)	2.02(1.19, 3.41) **	1.19(0.72, 1.97)	1.48(0.89, 2.43)	1.17(0.64, 2.12)	1.30(0.75, 2.27)	1.13(0.58, 2.21)
Merchant	1.29(0.68, 2.45)	1.10(0.56, 2.14)	1.10(0.59, 2.08)	2.38(1.27, 4.46)	1.35(0.65, 2.70)	1.02(0.51, 2.02)	0.54(0.23, 1.28)
**Monthly household income**							
< 1,368-birr	**1**	**1**	**1**	**1**	**1**	**1**	**1**
≥1,368-birr	0.37(0.21, 0.65) ***	0.39(0.19, 0.79) **	0.65(0.35, 1.21)	0.51(0.29, 0.87) *	0.04(0.01, 0.18) ***	0.11(0.03, 0.45) **	0.25(0.06, 1.09)
**Health facility at delivery**							
Health Center	1	1	1	1	1	1	1
Hospital	1.64(1.01, 2.66) *	1.96(1.22, 3.15) **	3.00(1.83, 4.91) ***	1.19(0.78, 1.80)	1.27(0.77, 2.12)	2.14(1.25, 3.69) **	1.72(0.86, 3.43)
**Presence of labor companion**							
**Yes**	**1**	**1**	**1**	**1**	**1**	**1**	**1**
NO	9.94(5.72, 17.28) ***	4.99(3.41, 7.32) ***	2.41(1.66, 3.50) ***	2.56(1.79, 3.66) ***	10.55(6.93, 16.06) ***	2.64(1.74, 3.99) ***	4.58(2.58, 8.15) ***
**No of delivery attendants**							
More than two	**1**	**1**	**1**	**1**	**1**	**1**	**1**
Two and less	0.71(0.45, 1.12)	1.01(0.68, 1.51)	0.77(0.53, 1.12)	0.96(0.66, 1.40)	0.73(0.47, 1.15)	0.83(0.54, 1.26)	0.76(0.45, 1.30)

*P≤ 0.05,

**P≤0.01,

***P≤0.001

Multi-collinearity and Pearson Correlation analyses (All variance inflation factor VIF less than 1.60). The R^2^ values of each model are described as follow: [Model 1 (All type of DA R2 = 30.5%), Model 2 (Physical abuse R2 = 26.5%), Model 3 (Non-dignified R2 = 14.2%), Model 4 (Non-consented R2 = 14.4%), Model 5 (Non-confidential R2 = 46.6%), Model 6 (Neglect/ Abandonment R2 = 19.3%) and Model 7 (Discrimination R2 = 15.9%)].

## Discussion

The findings of this study demonstrated that D&A during childbirth in public health facilities is a common experience among mothers in Western Ethiopia. Three fourths (95% CI: 71.28–78.16) of the women in this study reported experiences of D&A during childbirth. Findings from other sub-Saharan countries also reveal high rates of D&A during labor and delivery, for example, a study from Nigeria reported 98% of women experience D&A [10], while a study from Kenya reported 20% of women report D&A [6]. The high prevalence of D&A found in this study could potentially explain the low utilization of delivery care at health facilities in the study area and other similar settings.

More than 3 in 10 women reported experiences of physical abuse during delivery. A study from Nigeria reported 35.7% of women experienced physical abuse during childbirth [10]. Physical abuse (hitting and slapping) has been justified by providers in previous studies as a way to ‘put in line’ the ‘uncooperative and misbehaving’ woman in order to safely deliver and prevent distress of the baby [32]. In fact, women in labor are suffering themselves from the pain of childbirth, and rather than being supported, they are being mistreated. Government employees in this study were two times more likely to report physical abuse than women who are housewives. This may be explained by government employees being more aware of their rights, more sensitive to mistreatment, and very likely to report any form of D&A they faced. This implies that initiatives to empower women and help them understand their rights when visiting health facilities to seek care should target women who are less educated and not formally employed.

We found that experience with the health facility setting also matters in shaping perceptions of women who gave birth in the health facility. Women giving birth to their first child were about two times more likely to report experiencing D&A compared to women who had three or more children. While the former is unfamiliar with childbirth and with the way services are commonly provided at the health facilities, the latter may be familiar with the likelihood of experiencing D&A at the health facilities.

Unexpectedly, women who gave birth at a higher-level health facility, where quality of care is presumed to be better, had a greater likelihood of experiencing D&A. Women who delivered in hospitals were 1.6 times more likely to report any D&A and twice as likely to report physical abuse compared to women who delivered at a health center. Women who delivered in hospitals were also more likely to experience non-dignified care and neglect/abandonment compared to those who delivered at health centers, which could be the result of hospitals being understaffed with a high volume of patients, making it difficult for providers to adequately provide care. During the study period, our team witnessed that the study hospitals had three-delivery beds each and a maximum of five delivery attendants, which was far less than they needed. The frustration and burnout among the health care providers by the influx of women for delivery services in their constrained setting was evident. Regardless, the majority of women preferred hospitals over health centers for delivery because of a perception of better human resources and availability of supplies. As a result of the high demand, hospitals become overloaded and understaffed, exposing health care professionals to a poor working environment where frustration develops [26]. This, in turn, may increase the likelihood of inappropriate treatment of mothers by health professionals attending to the highly eventful and sensitive process of labor and delivery.

Although women are clearly experiencing high levels of D&A during delivery, it is common for a postpartum woman to think about her baby and her baby’s needs, feel relief from the pain that she experienced, and want to put the experiences of D&A behind her and move on. Other mothers who felt humiliated or mistreated and want to do something about it, often do not have a mechanism for redress, because legal frameworks for addressing medical malpractice and abuse during care are not common in most low-income countries [5]. As a result, complaints usually are ignored because there is no local system for accountability for D&A. Furthermore, health managers often do not consider or believe that the health professionals would commit D&A during delivery care with the intention of harming the women [5, 8].

Given that hospitals are preferred by clients over health centers in Ethiopia, an appropriate response to decrease D&A during delivery would be for policy makers and health planners to invest in expanding the size and skill mix of professionals in the preferred facilities (hospitals). This could possibly reduce the occurrence of D&A that may be occurring due to overcrowding, particularly if it is accompanied by intentional measures to sensitize health care providers and health managers regarding the magnitude and consequences of D&A.

Another common predictor for women to experience D&A was the absence of a companion in the laboring room with the women in labor. Women without a companion throughout labor and delivery were nearly ten times more likely to experience D&A than women with a companion. This indicates that providers are more likely cautious about how they act and speak to a client when a companion of the client is present, which suggests that the providers know that the way they behave in the absence of a companion is inappropriate [6]. The protective effect of having a companion with the mother during labor could also indicate that companions serve an important role as advocates for the laboring mother.

Permission to have a companion with mothers throughout labor and delivery is a work in progress in health facilities of the study area, as the Ministry of Health pushes for creating compassionate, caring and respectful healthcare professionals [27]. Allowing a family member or a relative to accompany a laboring woman not only minimizes the possibility of D&A by health professionals but will make mothers more comfortable while delivering in a health facility setting. Earlier works have also shown that presence of a companion throughout labor and delivery is a very effective strategy in minimizing D&A. Studies from Kenya, Tanzania and Ethiopia demonstrate that the presence of companion would reduce incidents of D&A [5, 6, 8, 25, 33]. Furthermore, presence of a companion has already been suggested for inclusion in the list of process indicators to monitor quality of childbirth care in the health facility [9].

Limitations of this study may include that the subjective reports of the women were not validated by objective observations of care. However, women’s own perceptions of experiences of D&A are ultimately what is most likely to influence their likelihood of repeating facility delivery. Furthermore, self-reported experiences of D&A have a critical effect on perceived quality of services provided at the health facility. A woman’s perception that she will receive respectful and friendly care will promote use of services when pregnant next time, and can also influence the perceptions of her social network.

This study addressed only the perspective of the clients and did not examine what the providers experience of attending deliveries in their particular context. Resource-limited countries suffer from structural, human and financial scarcity in the pursuit of providing quality healthcare services, which likely would have surfaced through including provider perspectives. We were however unable to address these aspects of the health system. This issue is vital because a previous study observed that health workers themselves face constraints to the provision of adequate health services, which affects their relationships with clients [34]. We would also like to note that our findings may not be generalizable to the situation of health facilities in other parts of Ethiopia. Furthermore, interpretation of the findings in this study should take note of the absence of standardized tools to measure D&A in the literature.

In conclusion, the findings of our study showed that D&A is a critical problem in the study area. Lack of a companion throughout labor and delivery services seems to expose women to a wide range of D&A. As such, having companion could largely contribute to ensuring the provision of respectful maternity care. Even though access to health care has improved dramatically, facilities persistently fail to provide respectful and friendly care to delivering women.

The spectrum of disrespectful and abusive experiences reported by the women is vast and calls for serious attention. To combat disrespectful and abusive treatment at health facilities, health managers and policymakers should prioritize enabling companions during labor and delivery, engage community members in health facility management boards, strengthen accountability through legal frameworks to appropriately address D&A, and improve the work environment for providers, coupled with training and introduction of care standards to professionals [23, 35, 36].

Adopting a patient-centered approach and strengthening health system resources directed towards maternity care could significantly improve the quality of care provided to delivering women. It is recommended that endeavors to promote birth at health facilities must address the issue of D&A to ensure higher utilization by women and to protect women’s fundamental rights during facility delivery. Possible interventions that could be designed and implemented at the health facility level include quality improvement interventions that focus on improving cultural responsiveness to mother’s preferences (e.g. birth companion and choice of birthing position). Addressing the unequal relationship between the women giving birth and skilled care providers is also a key area to addressing disrespectful care. Health facilities can also fight D&A by ensuring accountability (e.g. instruct and enforce desired standards of care, describing the nature of care that skilled providers and health facilities should offer to patients). Performance-based contracts and systems for providers and other health facility staff to report observed D&A by their peer providers represent another approach to promoting accountability [5]. Furthermore, the compassionate, respectful, caring workforce initiative of the government should address the critical role of resource availability at point of care, in addition to training of health professionals on ethical conduct and good practice [34].

Further research to uncover barriers and facilitators of respectful and dignified maternal care in relation to providers and the health system is needed to design and implement effective interventions to promote respectful maternity care in the context of limited resources.

## References

[1] Central Statistical Agency [Ethiopia], ORC Macro. 2006. Ethiopia Demographic and Health Survey 2005. Addis Ababa, Ethiopia and Calverton, Maryland, USA: Central Statistical Agency and ORC Macro.

[2] Central Statistical Agency [Ethiopia], ICF International. 2012. Ethiopia Demographic and Health Survey 2011. Addis Ababa, Ethiopia and Calverton, Maryland, USA: Central Statistical Agency and ICF International.

[3] Central Statistical Agency (CSA) [Ethiopia], ICF. 2016. Ethiopia Demographic and Health Survey 2016.: Addis Ababa, Ethiopia, and Rockville, Maryland, USA: CSA and ICF.

[4] Central Statistical Agency [Ethiopia]. 2014. Ethiopia Mini Demographic and Health Survey 2014.: Addis Ababa, Ethiopia.

[5] Bowser D, Hill K. Exploring evidence for disrespect and abuse in facility-based childbirth: report of a landscape analysis. USAID/TRAction Project. 2010.

[6] Abuya T, Warren CE, Miller N, Njuki R, Ndwiga C, Maranga A, et al Exploring the prevalence of disrespect and abuse during childbirth in Kenya. PLoS One. 2015;10(4):e0123606 10.1371/journal.pone.0123606 25884566 PMC4401776

[7] Banks KP, Karim AM, Ratcliffe HL, Betemariam W, Langer A. Jeopardizing quality at the frontline of healthcare: prevalence and risk factors for disrespect and abuse during facility-based childbirth in Ethiopia. Health Policy Plan. 2018;33(3):317–27. Epub 2018/01/09. 10.1093/heapol/czx180 29309598 PMC5886294

[8] Miller S, Lalonde A. The global epidemic of abuse and disrespect during childbirth: History, evidence, interventions, and FIGO′s mother− baby friendly birthing facilities initiative. International Journal of Gynecology & Obstetrics. 2015;131(S1). 10.1016/j.ijgo.2015.02.005 26433506

[9] Kruk ME, Gage AD, Arsenault C, Jordan K, Leslie HH, Roder-DeWan S, et al High-quality health systems in the Sustainable Development Goals era: time for a revolution. The Lancet Global Health. 2018;6(11):e1196–e252. 10.1016/S2214-109X(18)30386-3 30196093 PMC7734391

[10] Okafor II, Ugwu EO, Obi SN. Disrespect and abuse during facility‐based childbirth in a low‐income country. International Journal of Gynecology & Obstetrics. 2015;128(2):110–3. 25476154 10.1016/j.ijgo.2014.08.015

[11] Asefa A, Bekele D. Status of respectful and non-abusive care during facility-based childbirth in a hospital and health centers in Addis Ababa, Ethiopia. Reproductive health. 2015;12(1):33. 25890317 10.1186/s12978-015-0024-9 PMC4403719

[12] Sethi R, Gupta S, Oseni L, Mtimuni A, Rashidi T, Kachale F. The prevalence of disrespect and abuse during facility-based maternity care in Malawi: evidence from direct observations of labor and delivery. Reproductive health. 2017;14(1):111 10.1186/s12978-017-0370-x 28877701 PMC5588731

[13] Kea AZ, Tulloch O, Datiko DG, Theobald S, Kok MC. Exploring barriers to the use of formal maternal health services and priority areas for action in Sidama zone, southern Ethiopia. BMC pregnancy and childbirth. 2018;18(1):96 Epub 2018/04/14. 10.1186/s12884-018-1721-5 29649972 PMC5897996

[14] Bohren MA, Hunter EC, Munthe-Kaas HM, Souza JP, Vogel JP, Gülmezoglu AM. Facilitators and barriers to facility-based delivery in low-and middle-income countries: a qualitative evidence synthesis. Reproductive health. 2014;11(1):71 10.1186/1742-4755-11-71 25238684 PMC4247708

[15] King R, Jackson R, Dietsch E, Hailemariam A. Barriers and facilitators to accessing skilled birth attendants in Afar region, Ethiopia. Midwifery. 2015;31(5):540–6. Epub 2015/03/10. 10.1016/j.midw.2015.02.004 . 25745841

[16] Jalu MT, Ahmed A, Hashi A, Tekilu A. Exploring barriers to reproductive, maternal, child and neonatal (RMNCH) health-seeking behaviors in Somali region, Ethiopia. PLoS One. 2019;14(3):e0212227 Epub 2019/03/16. 10.1371/journal.pone.0212227 30875382 PMC6420011

[17] Bergen N, Abebe L, Asfaw S, Kiros G, Kulkarni MA, Mamo A, et al Maternity waiting areas—serving all women? Barriers and enablers of an equity-oriented maternal health intervention in Jimma Zone, Ethiopia. Glob Public Health. 2019:1–15. Epub 2019/03/25. 10.1080/17441692.2019.1597142 . 30905270

[18] Freedman LP, Kruk ME. Disrespect and abuse of women in childbirth: challenging the global quality and accountability agendas. The Lancet. 2014;384(9948):e42–e4. 10.1016/S0140-6736(14)60859-X 24965825

[19] Ishola F, Owolabi O, Filippi V. Disrespect and abuse of women during childbirth in Nigeria: a systematic review. PLoS One. 2017;12(3):e0174084 10.1371/journal.pone.0174084 28323860 PMC5360318

[20] Bohren MA, Vogel JP, Hunter EC, Lutsiv O, Makh SK, Souza JP, et al The mistreatment of women during childbirth in health facilities globally: a mixed-methods systematic review. PLoS medicine. 2015;12(6):e1001847 10.1371/journal.pmed.1001847 26126110 PMC4488322

[21] Abuya T, Ndwiga C, Ritter J, Kanya L, Bellows B, Binkin N, et al The effect of a multi-component intervention on disrespect and abuse during childbirth in Kenya. BMC pregnancy and childbirth. 2015;15(1):224. 26394616 10.1186/s12884-015-0645-6 PMC4580125

[22] d′Oliveira AFPL, Diniz SG, Schraiber LB. Violence against women in health-care institutions: an emerging problem. The Lancet. 2002;359(9318):1681–5. 10.1016/S0140-6736(02)08592-6 12020546

[23] Sadler M, Santos MJDS, Ruiz-Berdún D, Rojas GL, Skoko E, Gillen P, et al Moving beyond disrespect and abuse: addressing the structural dimensions of obstetric violence. Reproductive health matters. 2016;24(47):47–55. 10.1016/j.rhm.2016.04.002 27578338

[24] Wassihun B, Deribe L, Worede N, Gultie T. Prevalence of disrespect and abuse of women during child birth and associated factors in Bahir Dar town, Ethiopia. Epidemiology and health. 2018;40:e2018029 Epub 2018/07/31. 10.4178/epih.e2018029 30056644 PMC6178351

[25] Vogel JP, Bohren MA, Tunçalp Ӧ, Oladapo OT, Gülmezoglu AM. Promoting respect and preventing mistreatment during childbirth. BJOG: An International Journal of Obstetrics & Gynaecology. 2016;123(5):671–4. 26628382 10.1111/1471-0528.13750 PMC5063112

[26] Marine A, Ruotsalainen JH, Serra C, Verbeek JH. Preventing occupational stress in healthcare workers. Cochrane Database of Systematic Reviews. 2006;(4). 10.1002/14651858.CD002892.pub2 17054155

[27] Federal Democratic Republic of Ethiopia Ministry of H. HSTP Health Sector Transformation Plan 2015/16‐2019/20 (2008‐2012 EFY). Federal Democratic Republic of Ethiopia Ministry of Health; 2015.

[28] Federal Democratic Republic of Ethiopia Ministry of H. Health Sector Development Program IV, 2010/11–2014/15. Ministry of Health Addis Ababa; 2010.

[29] Rosen HE, Lynam PF, Carr C, Reis V, Ricca J, Bazant ES, et al Direct observation of respectful maternity care in five countries: a cross-sectional study of health facilities in East and Southern Africa. BMC pregnancy and childbirth. 2015;15(1):306. 26596353 10.1186/s12884-015-0728-4 PMC4657214

[30] Kruk ME, Kujawski S, Mbaruku G, Ramsey K, Moyo W, Freedman LP. Disrespectful and abusive treatment during facility delivery in Tanzania: a facility and community survey. Health Policy and Planning. 2014;33(1):e26–e33. 10.1093/heapol/czu079 29304252

[31] Sando D, Ratcliffe H, McDonald K, Spiegelman D, Lyatuu G, Mwanyika-Sando M, et al The prevalence of disrespect and abuse during facility-based childbirth in urban Tanzania. BMC pregnancy and childbirth. 2016;16(1):236. 27543002 10.1186/s12884-016-1019-4 PMC4992239

[32] Bohren MA, Vogel JP, Tunçalp Ö, Fawole B, Titiloye MA, Olutayo AO, et al “By slapping their laps, the patient will know that you truly care for her”: a qualitative study on social norms and acceptability of the mistreatment of women during childbirth in Abuja, Nigeria. SSM-population health. 2016;2:640–55. 10.1016/j.ssmph.2016.07.003 28345016 PMC5356417

[33] Sheferaw ED, Bazant E, Gibson H, Fenta HB, Ayalew F, Belay TB, et al Respectful maternity care in Ethiopian public health facilities. Reproductive health. 2017;14(1):60 10.1186/s12978-017-0323-4 28511685 PMC5434569

[34] Fonn S, Xaba M. Health Workers for Change: developing the initiative. Health policy and planning. 2001;16(suppl_1):13–8. 10.1093/heapol/16.suppl_1.13 11599664

[35] Burrowes S, Holcombe SJ, Jara D, Carter D, Smith K. Midwives′ and patients′ perspectives on disrespect and abuse during labor and delivery care in Ethiopia: a qualitative study. BMC pregnancy and childbirth. 2017;17(1):263 Epub 2017/08/24. 10.1186/s12884-017-1442-1 28830383 PMC5567643

[36] Ratcliffe HL, Sando D, Lyatuu GW, Emil F, Mwanyika-Sando M, Chalamilla G, et al Mitigating disrespect and abuse during childbirth in Tanzania: an exploratory study of the effects of two facility-based interventions in a large public hospital. Reproductive health. 2016;13(1):79 10.1186/s12978-016-0187-z 27424608 PMC4948096

